# Left Ventricular Longitudinal Dyssynchrony by CMR Feature Tracking Is Related to Adverse Prognosis in Advanced Arrhythmogenic Cardiomyopathy

**DOI:** 10.3389/fcvm.2021.712832

**Published:** 2021-10-11

**Authors:** Yanyan Song, Lu Li, Xiuyu Chen, Keshan Ji, Minjie Lu, Richard Hauer, Liang Chen, Shihua Zhao

**Affiliations:** ^1^Department of Magnetic Resonance Imaging, Fuwai Hospital, National Center for Cardiovascular Diseases, Chinese Academy of Medical Sciences and Peking Union Medical College, Beijing, China; ^2^Department of Diagnostic Radiology, National Cancer Center/National Clinical Research Center for Cancer/Cancer Hospital, Chinese Academy of Medical Sciences and Peking Union Medical College, Beijing, China; ^3^Netherlands Heart Institute and Department of Cardiology, University Medical Center, Utrecht, Netherlands; ^4^Department of Cardiac Surgery, Fuwai Hospital, National Center for Cardiovascular Diseases, Chinese Academy of Medical Sciences and Peking Union Medical College, Beijing, China

**Keywords:** arrhythmogenic cardiomyopathy, magnetic resonance imaging, feature tracking, dyssynchrony, prognosis

## Abstract

**Objectives:** Left ventricular (LV) involvement has been associated with unfavorable prognosis in arrhythmogenic cardiomyopathy (ACM). We aim to evaluate LV mechanics by cardiovascular magnetic resonance-feature tracking (CMR-FT) in ACM patients with right ventricular (RV) dysfunction.

**Methods:** We retrospectively recruited ACM patients diagnosed according to the revised Task Force Criteria (rTFC) from January 2015 to July 2017. All patients underwent CMR examinations and collections of clinical, electrocardiographic data. The strain and dyssynchrony parameters of LV and RV were analyzed. These patients were followed, and primary study outcome was defined as a composite of cardiovascular events (arrhythmic events and heart transplantation), secondary study outcome included arrhythmic events.

**Results:** Eighty-nine ACM patients (40.40 ± 13.98 years, 67.42% male) were included. LV and RV ejection fractions were 49.12 ± 12.02% and 22.28 ± 10.11%, respectively. During a median (IQR) follow-up for 18.20 (11.60-30.04) months, 30 patients experienced cardiovascular events which included 22 patients who experienced arrhythmic events. Patients with cardiovascular events had impaired LV global longitudinal strain (−10.82 ± 2.77 vs. −12.61 ± 3.18%, *p* = 0.010), impaired LV global circumferential strain (−11.81 ± 2.40 vs. −13.04 ± 2.83%, *p* = 0.044), and greater LV longitudinal dyssynchrony (LVLD) (80.98 ± 30.98 vs. 64.23 ± 25.51 ms, *p* = 0.012) than those without. After adjusting for age, sex, and other confounding factors, LVLD ≥89.15 ms was an independent risk factor for cardiovascular events (HR: 4.50, 95% CI: 1.94 to 10.42; *p* = 0.001) and for arrhythmic events (HR: 4.79, 95% CI: 1.74 to 13.20; *p* = 0.003).

**Conclusions:** LVLD by CMR-FT was an independent risk factor for cardiovascular and arrhythmic events in ACM patients in advanced stage, which could provide prognostic value for this subtype.

## Key Points

- Absence of prognostic value of conventional CMR parameters such as LVEF and RVEF.
- LV GLS ≥ −12.94% and LV GCS ≥ −13.11% were associated with cardiovascular events when adjusting for age and sex. However, it did not reach statistical significance after adjusting more confounders and in analysis of arrhythmic events.
- LVLD ≥ 89.15 ms assessed by CMR-FT was an independent risk factor for cardiovascular and arrhythmic events.

## Introduction

Arrhythmogenic right ventricular cardiomyopathy (ARVC) is an inheritable myocardial disease with potential high risk of malignant ventricular arrhythmias and progressive heart failure at end-stage (1, 2). It is classically characterized by fibro-fatty myocardial replacement predominantly at the right ventricle (RV) (3). Previously, left ventricular (LV) involvement has been observed exclusively in the end-stage of ARVC. More recently, LV involvement appeared to be present in the majority of ARVC patients, also in less advanced stages. In addition, a balanced biventricular and a left-side dominant phenotype has been identified (4, 5). Hence, the term arrhythmogenic cardiomyopathy (ACM) has recently been used to include different disease subcategories. Thus, ARVC, also described as classical ARVC, is a large predominant RV subcategory of ACM. However, all ACM subcategories are characterized by similar fibro-fatty alteration and life-threatening ventricular arrhythmias, usually already in the early disease stage (6). Heart failure and structural progression are also common and under-recognized in these patients (7). Numerous efforts have been made toward optimization of ACM risk stratification (6, 8–12). Detection of LV involvement is of clinical importance, which has been reported to provide incremental prognostic value (9, 13). However, the substantially predictive value of LV involvement could not be fully illustrated in the setting of ACM patients with heterogeneous severities of biventricular dysfunction.

Cardiac magnetic resonance (CMR) has emerged as an important tool in the evaluation of biventricular function due to its excellent myocardial-blood contrast and depiction of fibro-fatty tissue (14). Traditional CMR examination has been used for arrhythmic risk stratification in ACM-associated desmosomal mutation carriers (15). Current development of CMR feature-tracking (CMR-FT) techniques now enable quantification of ventricular mechanics from standard cine CMR images and has been introduced for evaluation of biventricular global, regional myocardial contraction and dyssynchrony (16–18). Furthermore, CMR-FT derived LV strain parameters have been identified as independent risk factors in a variety of cardiovascular diseases such as dilated cardiomyopathy and myocardial infarction (19–21). CMR-FT has been used for identification of preclinical ACM patients (22), and it has also been reported to detect a higher incidence of LV involvement even in ACM patients with preserved left ventricular ejection fraction (LVEF) (23, 24). However, the prognostic values of CMR-FT derived LV strain parameters in ACM population have rarely been studied (25). Therefore, this study aims to investigate the LV mechanics by CMR-FT and evaluate their prognostic values for cardiovascular and arrhythmic events in a subgroup of ACM patients in advanced stage.

## Methods

### Study Population

**Figure 1:**
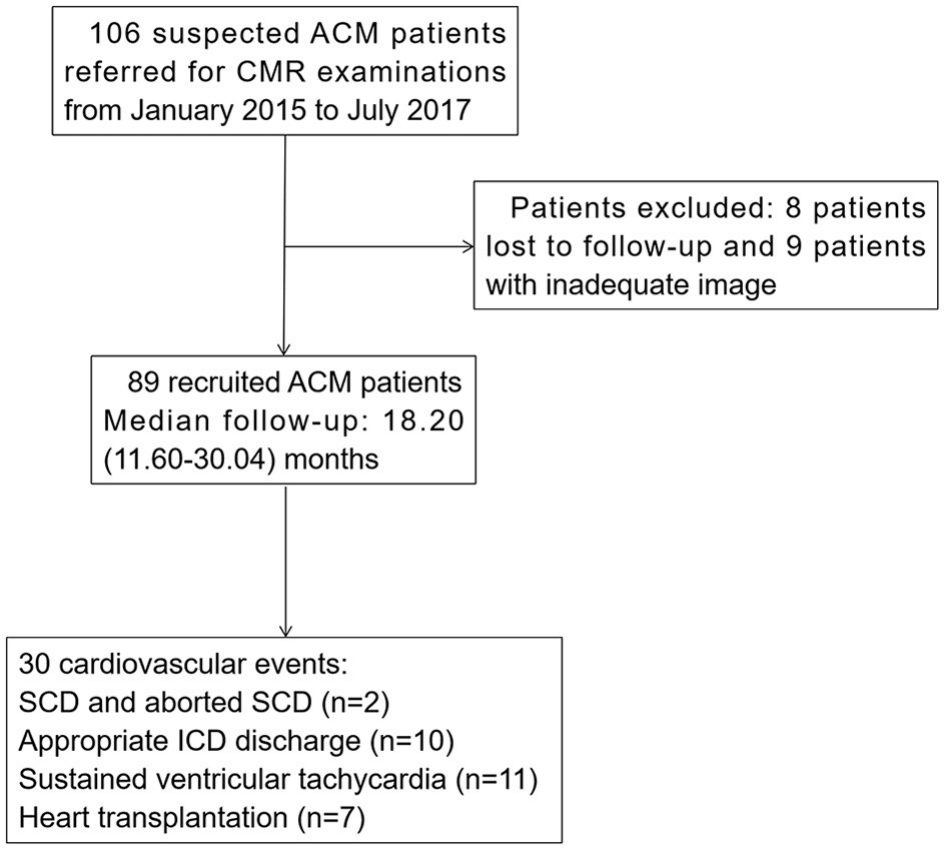
Flowchart of ACM patients' recruitment. ACM, arrhythmogenic cardiomyopathy; CMR, cardiac magnetic resonance; SCD, sudden cardiac death; ICD, implantable cardioverter-defibrillator.

We retrospectively reviewed 106 patients ≥ 15 years old with suspected ACM referred for CMR examinations from January 2015 to July 2017 at Fuwai Hospital. Demographics, clinical information, 12-lead electrocardiography (ECG), and 24-h Holter monitoring data were collected from the medical records within 2 weeks of CMR examinations. The diagnostic standards for ACM were based on the revised task force criteria (rTFC) score with either two major criteria, one major and two minor criteria, or four minor criteria (26). All patients were followed up from the initiation of CMR examination to the most recent evaluation by clinical visit or telephone review, or to the latest cardiovascular events. Eight patients lost to follow-up and nine patients with inadequate image quality were excluded. Finally, 89 consecutive ACM patients were enrolled in this study (Figure 1). This study was approved by the hospital institutional review board and informed consents were obtained from all patients.

All patients were followed up via clinic visit, medical recording, or telephone interview (every 6 months). The primary study outcome was defined as a composite of cardiovascular events including heart transplantation, sudden cardiac death (SCD), resuscitated cardiac arrest, sustained ventricular tachycardia (SVT) (ventricular tachycardia lasting >30 s at >100 b.p.m. or with hemodynamic compromise requiring cardioversion), ventricular fibrillation/flutter, and appropriate implantable cardioverter-defibrillator (ICD) discharge. Appropriate ICD discharge was defined as appropriate if triggered by life-threatening arrhythmias: ventricular tachycardia above the programmed cutoff of the ICD (12 intervals at >180/min) or ventricular fibrillation. The secondary study outcome was defined as arrhythmic events including SCD, SVT, ventricular fibrillation/flutter, and appropriate ICD discharge.

### Cardiovascular Magnetic Resonance

CMR scans were performed on a 3-T scanner (Discovery MR750W, GE Healthcare, Milwaukee, WI) and 3-T scanner (Ingenia, Philips Healthcare, Best, The Netherlands) with a phased-array cardiovascular coil and electrocardiographic respiratory gating. All sequences were acquired with breath holding. Three single long-axis cine images (LV two-chamber, four-chamber, and LV outflow tract) and 8 short-axis slices from the base of the mitral valve to the apex were acquired using balanced steady state free precession sequence (b-SSFP). Typical imaging parameters were as follows: field of view (FOV) = 320 × 320 mm, matrix = 192 × 224, repetition time (TR) = 3.3 ms, echo time (TE) = 1.7 ms, flip angle = 50°, number of cardiac frames = 25 per cardiac cycle, slice thickness = 8 mm, slice gap = 2 mm. Fat- and non-fat-suppressed fast spin-echo sequences were acquired identical with mid short-axis and LV four-chamber images with double-inversion recovery blood suppression pulses. Typical imaging parameters were as follows: FOV = 320 × 320 mm, matrix = 192 × 224, TR = 1-2 R-R intervals, TE = 10 ms, slice thickness = 8 mm, slice gap = 2 mm. The LGE images were acquired 10-15 min after intravenously injected gadolinium-DTPA (Magnevist, Schering AG, Berlin, Germany; 0.2 mmol/kg) in identical long-axis and short-axis planes using segmented phase-sensitive inversion recovery (PSIR) sequence. Typical imaging parameters were: FOV = 380 × 320 mm, matrix = 256 × 162, TR = 8.6 ms, TE = 3.36 ms, flip angle = 25°, slice thickness = 8 mm, slice gap = 2 mm, nominal TI = 300-350 ms.

### Conventional CMR Analysis

Biventricular functions were analyzed using CVI42 (version 5.0, Circle Cardiovascular Imaging Inc., Calgary, Canada) by two radiologists with 8 years and 10 years of experience in CMR post-processing, who were blinded to clinical data. The endocardial and epicardial contours of both ventricles were manually traced at end-diastole and end-systole on 8 short-axis cine image slices. Papillary muscles were excluded from calculation of volumes. End-diastolic volume (EDV), end-systolic volume (ESV), and ejection fraction for both ventricles were generated automatically. All volumetric measurements were indexed to body surface area (BSA). Two, four chamber and eight short-axis LGE images were visually inspected by two independent observers to determine the presence of LGE in LV with excellent contrast between enhanced (bright) and normal myocardium (black). And the discrepancies between the two readers were adjudicated by a senior observer. The number of LGE segments in LV myocardium was also calculated according to the American Heart Association (AHA) 17-segment model.

### Feature Tracking Analysis

The CMR FT analysis was performed on the acquired cine images using CVI42 (version 5.0, Circle Cardiovascular Imaging Inc., Calgary, Canada). End diastolic endo- and epicardial contours were traced semi-automatically in long-axis views (two-chamber, three-chamber, and four-chamber) and short-axis view on cine images by investigators blinded to the clinical and CMR data. Adjustments were made after visual inspection during cine loop playback to ensure appropriate tracking of LV segments. For LV strain parameters, three long-axis and short-axis views of cine images were used to assess global and regional (basal, mid, and apical) peak strain in longitudinal, circumferential, and radial directions. The LV segmental strain parameters were provided according to the American Heart Association 16-segment model (24). In addition, the LV longitudinal, circumferential, and radial dyssynchrony was defined as the standard deviation (SD) of the time-to-peak strain in all LV segments. For RV strain parameters, a 4-chamber view of cine images was used to obtain RV global longitudinal peak strain and short-axis views of cine images were used to obtain RV global circumferential and radial peak strain. RV circumferential and radial dyssynchrony was defined as the standard deviation (SD) of the time-to-peak strain in all RV segments. RV longitudinal dyssynchrony was not analyzed in this study due to inadequate RV algorithm by software for it was measured only in a 4-chamber view of cine images.

### Statistical Analysis

All continuous variables were given as mean ± SD or as median values with interquartile range if normally distributed. Categorical variables were presented as percentages. The chi-square test or Fisher's exact test was used for comparisons of categorical variables, as appropriate. Student's *t*-test were performed for comparisons of normally distributed continuous variables. Non-parametric tests were performed using the Mann-Whitney *U*-test. Univariate and multivariate Cox proportional hazards regression analysis were used to calculate the hazard ratios (HR) and 95% confidence intervals (CI) of risk factors. The multivariable model was constructed to adjust for possible confounders with: (1) *p*-value < 0.1 in the univariate model; or (2) risk factors reported in previous studies. Receiver operating characteristics (ROC) analysis was applied to define the optimal cut-off values for dichotomizing continuous risk markers. Kaplan-Meier analyses of estimated event-free survival for the risk factors were conducted with log-rank test. The intra-class correlation coefficient (ICC) analysis was used to assess the inter- and intra-observer variability for biventricular strain parameters. A two-sided *p*-value of <0.05 was considered statistically significant. All the analyses were performed with the statistical software packages R (http://www.R-project.org, The R Foundation) and EmpowerStats (http://www.empowerstats.com, X&Y Solution, Inc., Boston, MA).

## Results

### Baseline Characteristics

Eighty-nine ACM patients, aged 40.40 ± 13.98 years, 67.42% male, were recruited in this study. The baseline characteristics of the patients were presented in Table 1. NYHA III-IV class was present in 15 (16.85%) individuals. Twenty-one (23.60%) patients had RBBB, and none of patients had LBBB. During follow-up for 18.20 (11.60-30.04) months, 30 patients reached end-point events, including: SCD and aborted SCD (*n* = 2), appropriate ICD discharge (*n* = 10), sustained ventricular tachycardia (*n* = 11), and heart transplantation (*n* = 7). Four of the seven heart transplantation patients experienced sustained ventricular arrhythmias prior to surgery. All patients were further divided into patients with (*n* = 30) or without (*n* = 59) events. Patients with events had a higher proportion of recent syncope (<6 months) [40.00 vs. 20.34%, *p* = 0.048] than that in the non-event group. No other significant differences were observed in terms of baseline characteristics among the two groups.

**Table 1:** Baseline clinical characteristics of ACM population and comparison of patients without and with cardiovascular events during follow-up.

	**All (*n* = 89)**	**No CE (*n*=59)**	**CE (*n* =30)**	***P*-value**
Age at diagnosis (y)	40.40 ±13.98	40.02 ± 13.72	41.17 ± 14.69	0.716
Male gender, *n* (%)	60 (67.42%)	41 (69.49%)	19 (63.33%)	0.558
NYHA class, *n* (%)				0.145
I	24 (26.97%)	18 (30.51%)	6 (20.00%)	
II	50 (56.18%)	34 (57.63%)	16 (53.33%)	
III	8 (8.99%)	5 (8.47%)	3 (10.00%)	
IV	7 (7.87%)	2 (3.39%)	5 (16.67%)	
Recent cardiac syncope, *n* (%)	24 (26.97%)	12 (20.34%)	12 (40.00%)	0.048
Family history of ACM, *n* (%)	13 (14.61%)	6 (10.17%)	7 (23.33%)	0.096
History of SVT	38 (42.70%)	21 (35.59%)	17 (56.67%)	0.057
Major repolarization criterion, *n* (%)	37 (41.57%)	25 (42.37%)	12 (40.00%)	0.830
TWI in ≥3 precordial leads	63 (70.79%)	40 (67.80%)	23 (76.67%)	0.384
TWI in ≥2 inferior leads	27 (30.34%)	19 (32.20%)	8 (26.67%)	0.591
Number of TWI, *n* (median)	4.00 (3.00-6.00)	4.00 (3.00-6.00)	5.00 (3.00-6.00)	0.488
Major depolarization criterion, *n* (%)	4 (4.49%)	3 (5.08%)	1(3.33%)	1.000
Arrhythmias major criterion, *n* (%)	27 (30.34%)	15 (25.42%)	12 (40.00%)	0.157
rTFC score, *n* (median)	5.00 (4.00-6.00)	4.00 (4.00-5.00)	6.00 (4.00-6.00)	<0.001
NSVT, *n* (%) (*n* = 55)	18 (32.73%)	6 (27.30%)	12 (36.40%)	0.481
24 h PVC count, *n* (%) (*n* = 55)	1,904 (344-5,681)	1,904 (159-5,662)	2,125 (443-7,435)	0.830
RBBB, *n* (%)	21 (23.60%)	16 (27.12%)	5 (16.67%)	0.272
LBBB, *n* (%)	0	0	0	1.000
First degree AV block, *n* (%)	6 (6.74%)	4 (6.78%)	2 (6.67%)	1.000
Therapy, *n* (%)				
Beta-blockers	60 (67.42%)	36 (61.02%)	24 (80.00%)	0.071
ACE inhibitors	47 (52.81%)	30 (50.85%)	17 (56.67%)	0.603
Antiarrhythmic drug	42 (47.19%)	27 (45.76%)	15 (50.00%)	0.705
Diuretic agent	24 (26.97%)	15 (25.42%)	9 (30.00%)	0.646
ICD	12 (13.48%)	6 (10.17%)	6 (20.00%)	0.199
Radiofrequency ablation	33 (37.08%)	19 (32.20%)	14 (46.67%)	0.182

*Data were presented as percentages in parentheses, means ± standard deviations or median values with interquartile range in parentheses*.

*ACE, angiotensin converting enzyme; ACM, arrhythmogenic cardiomyopathy; AV, atrio-ventricular; CE, cardiovascular events; ICD, implantable cardioverter defibrillator; LBBB, left bundle branch block; NSVT, non-sustained ventricular tachycardia; NYHA, New York Heart Association; PVC, premature ventricular complexes; RBBB, right bundle branch block; rTFC, revised Task Force Criteria; SVT, sustained ventricular tachycardia; TWI, T wave inversion*.

### Conventional CMR and CMR-FT Characteristics

As shown in Table 2, the average values of LVEF and RVEF were 49.12 ± 12.02% and 22.28 ± 10.11%, respectively, suggesting an advanced stage of ACM in our cohort. Patients with events had larger LVEDVi (79.97 ± 22.65 vs. 71.02 ± 20.02 ml/m^2^, *p* = 0.054) compared with patients without events, while there were no other significant differences between the two groups in terms of conventional CMR parameters.

**Table 2:** Conventional CMR parameters of ACM population and comparison of patients without and with cardiovascular events during follow-up.

	**All (*n* = 89)**	**No CE (*n* = 59)**	**CE (*n* = 30)**	***P*-value**
LVEF (%)	49.12 ± 12.02	50.47 ± 10.98	46.46 ± 13.66	0.137
LVEF <50 (%)	38 (42.70%)	23 (38.98%)	15 (50.00%)	0.321
LVEDVi (ml/m^2^)	74.04 ± 21.25	71.02 ± 20.02	79.97 ± 22.65	0.054
LVESVi (ml/m^2^)	39.01 ± 19.35	36.24 ± 16.53	44.44 ± 23.32	0.100
LV fat infiltration, *n* (%)	37 (41.57%)	28 (47.46%)	9 (30.00%)	0.114
LV WMA, *n* (%)	21 (23.60%)	14 (23.73%)	7 (23.33%)	0.967
LV LGE, *n* (%)	54 (57.95%)	35 (59.32%)	19 (63.33%)	0.714
LV LGE extent (%) (*n* = 54)	11.92 (8.42-19.07)	11.56 (7.57-17.30)	14.30 (9.10-22.20)	0.273
LV involvement by CMR, *n* (%)	65 (73.03%)	43 (72.88%)	22 (73.33%)	0.964
RVEF (%)	22.28 ± 10.11	22.07 ± 10.42	22.69 ± 9.62	0.789
RVEF <40 (%)	85 (95.51%)	55 (93.22%)	30 (100.00%)	0.144
RVEDVi (ml/m^2^)	129.03 ± 53.28	122.17 ± 48.50	142.50 ± 60.21	0.216
RVESVi (ml/m^2^)	102.50 ± 50.84	97.26 ± 46.51	112.80 ± 57.90	0.381
RV fat infiltration	45 (50.56%)	29 (49.15%)	16 (53.33%)	0.709
RV WMA, *n* (%)	28 (31.46%)	20 (33.90%)	8 (26.67%)	0.487
RV LGE, *n* (%)	89(100)	59(100)	30(100)	1.000

*Data were presented as means ± standard deviations or median values with interquartile range in parentheses*.

*ACM, arrhythmogenic cardiomyopathy; CE, cardiovascular events; CMR, cardiac magnetic resonance; EDVi, end-diastolic volume index; EF, ejection fraction; ESVi, end-systolic volume index; LGE, late gadolinium enhancement; LV, left ventricular; RV, right ventricular; WMA, wall motion abnormality*.

**Figure 2:**
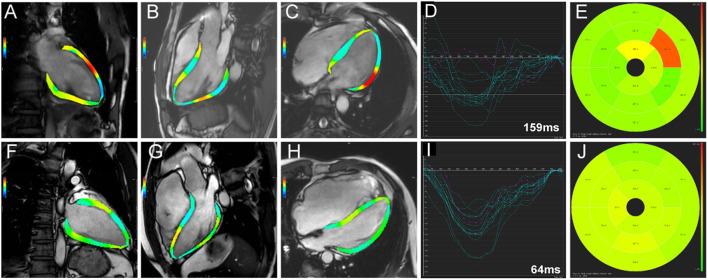
Representative cases from patients with **(A-E)** and without events **(F-J)** group. Two-, three-, and four-chamber views of left ventricular strain calculated by software were presented in **(A,F)**, **(B,G)**, and **(C,H)**, respectively. The time-independent left ventricular longitudinal strain curves of American Heart Association 16 segments were shown in **(D,I)**, respectively. LV longitudinal dyssynchrony was 159 ms in patients with event **(D)** and 64 ms in patients without event **(I)** by quantification. The time-to-peak longitudinal strain of the 16 segments was demonstrated in **(E,J)**, respectively.

Compared with the non-event group, patients with events had significantly impaired LV GLS (−10.82 ± 2.77% vs. −12.61 ± 3.18%, *p* = 0.010), impaired LV GCS (−11.81 ± 2.40% vs. −13.04 ± 2.83%, *p* = 0.044), and greater LVLD (80.98 ± 30.98 vs. 64.23 ± 25.51 ms, *p* = 0.012) in CMR-FT analysis (Table 3). Besides, in the further subgroup analysis, patients with events had significantly impaired LV mid and apical longitudinal strain compared with patients without events (*p* = 0.015 and *p* = 0.021, respectively). Representative cases from patients with and without events were shown in Figure 2. No significant differences were observed in RV dyssynchrony and strain parameters between the two groups (Table 3).

**Table 3:** CMR-FT parameters of ACM population and comparison of patients without and with cardiovascular events during follow-up.

	**All (*n* = 89)**	**No CE (*n* = 59)**	**CE (*n* = 30)**	***P*-value**
**Dyssynchrony parameters**				
LVLD (ms)	69.88 ± 28.44	64.23 ± 25.51	80.98 ± 30.98	0.012
LVCD (ms)	69.08 ± 31.94	66.33 ± 26.62	74.40 ± 40.30	0.264
LVRD (ms)	79.89 ± 46.27	74.04 ± 29.21	91.40 ± 67.71	0.094
RVCD (ms)	123.86 ± 88.24	116.75 ± 83.54	137.85 ± 96.78	0.284
RVRD (ms)	109.57 ± 84.39	104.83 ± 77.16	118.89 ± 97.82	0.460
**LV longitudinal strain**				
Global (%)	−12.01 ± 3.15	−12.61 ± 3.18	−10.82 ± 2.77	0.010
Basal (%)	−8.80 ± 3.85	−9.05 ± 4.48	−8.29 ± 2.09	0.381
Mid (%)	−12.06 ± 5.26	−12.85 ± 5.70	−10.50 ± 3.89	0.015
Apical (%)	−11.44 ± 4.24	−12.10 ± 4.40	−10.14 ± 3.64	0.021
**LV circumferential strain**				
Global (%)	−12.63 ± 2.74	−13.04 ± 2.83	−11.81 ± 2.40	0.044
Basal (%)	−11.72 ± 4.79	−12.16 ± 3.90	−10.86 ± 6.17	0.227
Mid (%)	−13.42 ± 4.39	−14.08 ± 4.30	−12.13 ± 4.36	0.048
Apical (%)	−13.29 ± 5.31	−13.53 ± 5.35	−12.82 ± 5.27	0.558
**LV radial strain**				
Global (%)	32.00 ± 9.61	32.90 ± 9.28	30.25 ± 10.15	0.221
Basal (%)	32.23 ± 11.97	33.70 ± 12.60	29.33 ± 10.21	0.104
Mid (%)	25.34 ± 10.16	26.21 ± 9.50	23.64 ± 11.31	0.262
Apical (%)	32.11 ± 13.57	33.73 ± 14.15	28.93 ± 11.95	0.099
**RV strain parameters**				
GLS (%)	−11.78 ± 6.38	−11.78 ± 6.97	−11.77 ± 5.15	0.993
GCS (%)	−4.54 ± 4.52	−4.79 ± 4.35	−4.05 ± 4.89	0.612
GRS (%)	9.97 ± 6.39	10.42 ± 6.36	9.09 ± 6.48	0.284

*Data were presented as means ± standard deviations or median values with interquartile range in parentheses*.

*ACM, arrhythmogenic cardiomyopathy; CE, cardiovascular events; CMR, cardiac magnetic resonance; FT, feature tracking; LV, left ventricular; RV, right ventricular; LD, longitudinal dyssynchrony; CD, circumferential dyssynchrony; RD, radial dyssynchrony; GCS, global circumferential strain; GLS, global longitudinal strain; GRS, global radial strain*.

### Relationship Between CMR-FT Parameters and Events

**Figure 3:**
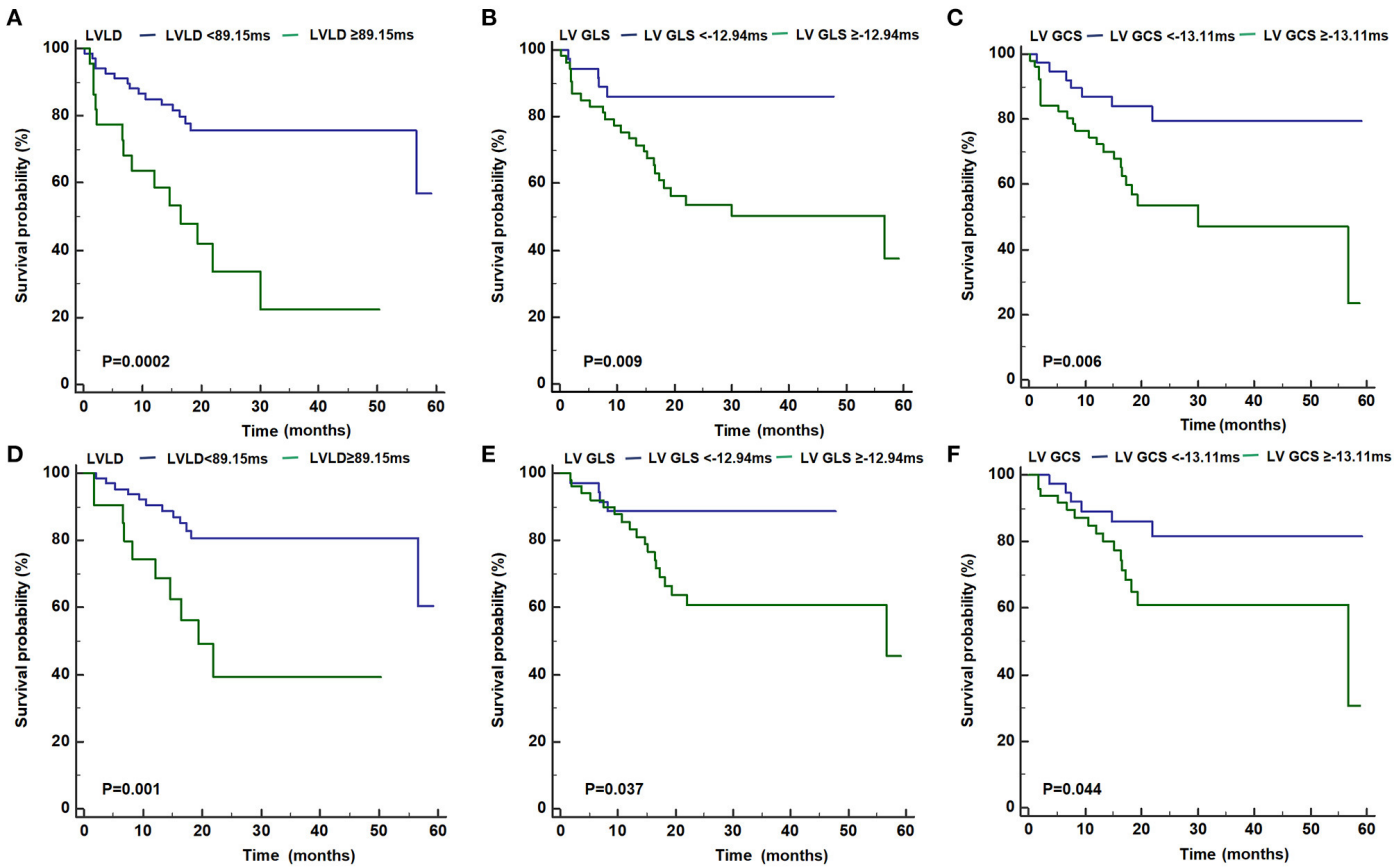
Kaplan-Meier curves for the primary **(A–C)** and secondary **(D–F)** outcome for patients with LVLD ≥ 89.15 ms vs. with LVLD < 89.15 ms, with LV GLS ≥ −12.94% vs. with LV GLS <−12.94%, and with GCS ≥ −13.11% vs. with LV GCS < −13.11%, respectively. ACM, arrhythmogenic cardiomyopathy; GLS, global longitudinal strain; GCS, global circumferential strain; LV, left ventricular; LVLD, LV longitudinal dyssynchrony.

As shown in Figure 3, patients with LVLD ≥ 89.15 ms had worse outcomes than those with LVLD < 89.15 ms for cardiovascular events (log rank *p* = 0.0002) and arrhythmic events (log rank *p* = 0.001). Patients with LV GLS ≥ −12.94% had worse survival free from cardiovascular events than those with LV GLS < −12.94% (log rank *p* = 0.009) and arrhythmic events (log rank *p* = 0.037). Patients with LV GCS ≥ −13.11% had worse survival free from cardiovascular events than those with LV GLS < −13.11% (log rank *p* = 0.006) and arrhythmic events (log rank *p* = 0.044). Moreover, we performed univariate and multivariate Cox proportional hazards analysis for LVLD, LV GLS, and LV GCS as shown in Tables 4, 5. It was shown that LVLD was significantly associated with cardiovascular events (HR: 1.02, 95% CI: 1.00-1.04; *p* = 0.002) and arrhythmic events (HR: 1.03, 95% CI: 1.01-1.05; *p* = 0.002) even after adjusting for confounding variables (age, sex, syncope, SVT history, beta-blockers, family history of ACM, number of TWI, major repolarization criterion, LVEF, and LVEDVi). When included as a categorical variable, LVLD ≥ 89.15 ms was an independent risk factor for cardiovascular events (HR: 4.50, 95% CI: 1.94-10.42; *p* = 0.001) and arrhythmic events (HR: 4.79, 95% CI: 1.74-13.20; *p* = 0.003) after adjusting for the above confounding variables. LV GLS ≥ −12.94% and LV GCS ≥ −13.11% was associated with cardiovascular events (HR: 3.45, 95% CI: 1.31-9.09, *p* = 0.012; HR: 3.28, 95% CI: 1.39-7.74, *p* = 0.007, respectively) and arrhythmic events (HR: 3.00, 95% CI: 1.01-8.95, *p* = 0.048; HR: 2.55, 95% CI: 0.99-6.58, *p* = 0.053, respectively) adjusting for age and sex. However, it did not reach statistical significance after adjusting more confounders in model II.

**Table 4:** Multivariate Cox proportional hazards analysis of cardiovascular events in ACM population.

**Variable**	**Non-adjusted**	***P*-value**	**Model I**	***P*-value**	**Model II**	***P*-value**
LVLD (ms)	1.02 (1.00, 1.03)	0.004	1.02 (1.01, 1.03)	0.003	1.02 (1.01, 1.04)	0.002
LVLD classification						
<89.15	Reference		Reference		Reference	
≥89.15	3.67 (1.77, 7.63)	0.001	4.19 (1.96, 8.95)	0.002	4.50 (1.94, 10.42)	0.001
LV GLS (%)	1.12 (1.00, 1.26)	0.056	1.14 (1.00, 1.28)	0.044	1.02 (0.86, 1.20)	0.837
LV GLS classification						
< −12.94	Reference		Reference		Reference	
≥−12.94	3.34 (1.27, 8.75)	0.014	3.45 (1.31, 9.09)	0.012	1.68 (0.54, 5.22)	0.371
LV GCS (%)	1.18 (1.03, 1.35)	0.017	1.19 (1.04, 1.37)	0.013	1.18 (0.96, 1.45)	0.119
LV GCS classification						
< −13.11	Reference		Reference		Reference	
≥−13.11	3.08 (1.32, 7.21)	0.010	3.28 (1.39, 7.74)	0.007	2.30 (0.90, 5.89)	0.082

*Model I adjust for: age and sex*.

*Model II adjust for: age, sex, syncope, sustained ventricular tachycardia history, beta-blockers, family history of ACM, number of T wave inversion, major repolarization criterion, left ventricular ejection fraction, left ventricular end-diastolic volume index*.

*ACM, arrhythmogenic cardiomyopathy; LV, left ventricular; LVLD, left ventricular longitudinal dyssynchrony; GLS, global longitudinal strain; GCS, global circumferential strain*.

**Table 5:** Multivariate Cox proportional hazards analysis of arrhythmic events in ACM population.

**Variable**	**Non-adjusted**	***P*-value**	**Model I**	***P*-value**	**Model II**	***P*-value**
LVLD (ms)	1.02 (1.00, 1.03)	0.014	1.02 (1.00, 1.03)	0.015	1.03 (1.01, 1.05)	0.002
LVLD classification						
<89.15	Reference		Reference		Reference	
≥89.15	3.70 (1.57, 8.72)	0.003	4.02(1.65, 9.81)	0.002	4.79 (1.74, 13.20)	0.003
LV GLS (%)	1.10 (0.96, 1.26)	0.182	1.10 (0.95, 1.27)	0.187	1.10 (0.91, 1.35)	0.327
LV GLS classification						
< −12.94	Reference		Reference		Reference	
≥−12.94	3.01 (1.01, 8.95)	0.047	3.00 (1.01, 8.95)	0.048	2.00 (0.56, 7.21)	0.287
LV GCS (%)	1.02 (0.87, 1.19)	0.833	1.02 (1.87, 1.19)	0.842	1.04 (0.82, 1.34)	0.728
LV GCS classification						
< −13.11	Reference		Reference		Reference	
≥−13.11	2.54 (0.99, 6.52)	0.052	2.55 (0.99, 6.58)	0.053	2.49 (0.85, 7.33)	0.097

*Model I adjust for: age and sex*.

*Model II adjust for: age, sex, syncope, sustained ventricular tachycardia history, beta-blockers, family history of ACM, number of T wave inversion, major repolarization criterion, left ventricular ejection fraction, left ventricular end-diastolic volume index*.

*ACM, arrhythmogenic cardiomyopathy; LV, left ventricular; LVLD, left ventricular longitudinal dyssynchrony; GLS, global longitudinal strain; GCS, global circumferential strain*.

### Inter-Observer and Intra-Observer Variability

The inter- and intra-observer variability for biventricular strain parameters are summarized in Table 6. All CMR-FT derived strain parameters showed good to excellent intra-observer (0.82-0.95) and inter-observer (0.80-0.91) variability.

**Table 6:** Intra-observer and inter-observer reproducibility for CMR-FT derived strain parameters.

	**Intra-observer**		**Inter-observer**	
	**ICC**	**95% CI**	**ICC**	**95% CI**
**Dyssynchrony parameters**				
LVLD (ms)	0.92	0.84-0.96	0.89	0.81-0.94
LVCD (ms)	0.89	0.80-0.96	0.84	0.75-0.92
LVRD (ms)	0.83	0.68-0.94	0.80	0.65-0.85
RVCD (ms)	0.86	0.74-0.91	0.83	0.65-0.88
RVRD (ms)	0.82	0.67-0.89	0.80	0.59-0.86
**LV longitudinal strain**				
Global (%)	0.95	0.88-0.97	0.91	0.80-0.96
Basal (%)	0.91	0.80-0.95	0.85	0.74-0.90
Mid (%)	0.90	0.80-0.93	0.88	0.77-0.92
Apical (%)	0.89	0.76-0.93	0.83	0.70-0.93
**LV circumferential strain**				
Global (%)	0.93	0.86-0.98	0.90	0.81-0.95
Basal (%)	0.90	0.76-0.96	0.86	0.73-0.91
Mid (%)	0.92	0.81-0.95	0.85	0.71-0.92
Apical (%)	0.87	0.77-0.90	0.83	0.69-0.90
**LV radial strain**				
Global (%)	0.91	0.79-0.96	0.85	0.68-0.95
Basal (%)	0.87	0.73-0.92	0.83	0.67-0.91
Mid (%)	0.89	0.75-0.94	0.84	0.70-0.93
Apical (%)	0.85	0.71-0.93	0.82	0.65-0.90
**RV strain parameters**				
GLS (%)	0.89	0.80-0.94	0.87	0.79-0.92
GCS (%)	0.90	0.78-0.96	0.86	0.71-0.94
GRS (%)	0.87	0.69-0.96	0.81	0.62-0.89

*CI, confidence interval; CMR, cardiac magnetic resonance; FT, feature tracking; ICC, intraclass correlation coefficient; LV, left ventricular; RV, right ventricular; LD, longitudinal dyssynchrony; CD, circumferential dyssynchrony; RD, radial dyssynchrony; GCS, global circumferential strain; GLS, global longitudinal strain; GRS, global radial strain*.

## Discussion

In the present study, we introduced CMR-FT technique in evaluating LV and RV mechanics in a sizable definite ACM cohort, in which all patients were recruited in a tertiary referral center and had advanced RV dysfunction. Thus, we underlined the prognostic value of LV mechanics in the setting of homogeneous RV functional status, which was different from previously reported study cohorts. In the absence of identified conventional risk stratification parameters, the present study showed LVLD ≥ 89.15 ms assessed by CMR-FT was an independent risk factor for cardiovascular and arrhythmic events. The prognostic value of LV GLS and LV GCS was less confirmed in this study cohort.

In this study, we included patients with more advanced stages of RV dysfunction compared with other Western ACM populations (8, 9, 27). Cadrin-Tourigny et al. (8) reported that 27.7% of ACM patients experienced life-threatening ventricular arrhythmia and 14 (2.7%) patients underwent heart transplantation in 528 definite ACM patients. Lie et al. (27) showed that 18 (15%) patients experienced life-threatening ventricular arrhythmias and only one patient experienced heart transplantation in 117 ACM probands and mutation-positive family members. For both studies, the median follow-up duration was more than 4 years. However, in this study, 23 (25.84%) patients experienced arrhythmic events and 7 (7.87%) patients experienced heart transplantation during a short median follow-up for 18.20 months, implying an advanced disease status in this study cohort.

The presence of LV dysfunction in ACM patients is of clinical significance. Several studies have emphasized the incremental prognostic value of LV involvement in the risk stratification of ACM patients (9, 13, 28, 29). LVEF was irrelevant of cardiovascular events in our ACM population, which was also in accordance with other studies (8, 27, 30). However, patients with events had no differences with those without events in terms of LV involvement by CMR, which was different from Aquaro et al.'s study (29). They recruited more early stage ACM patients (LVEF 57 ± 12%,RVEF 53 ± 13%) including CMR negative patients, which could explain the differences. LV ventricular mechanical parameters derived from echocardiography have been proposed in evaluation of LV dysfunction (31). Mast et al. (29) revealed LV involvement in 68% of ACM patients and their relatives by echocardiographic deformation imaging. In addition, this technique appeared to be an independent prognostic marker of composite cardiovascular events (32). Lie et al. (27) reported that LV longitudinal dyssynchrony assessed by echocardiography was a strong risk marker for arrhythmic events in consecutive ACM probands and mutation-positive family members. By virtue of excellent myocardial-blood contrast and depiction of fibro-fatty tissue, CMR has been used for diagnosis and risk stratification of ACM patients and mutation carriers (15). CMR-FT technique has provided a novel tool for evaluation of LV myocardial strain and dyssynchrony. In comparison with echocardiographic speckle tracking, CMR-FT has superior spatial resolution for reliable tracking of myocardium, may be less operator dependent, and can be applied on routine cine CMR images. The feasibility of CMR-FT technique has been validated in comparison to CMR tagging or echocardiographic speckle tracking (17, 18). In addition, CMR-FT has been used for identification of preclinical ACM patients with preserved left ventricular ejection fraction (LVEF) (22, 23) and for risk stratification in ACM (25). Recently, Shen et al. (25) reported that CMR-FT derived LV GLS > −12.65% was an independent risk factor for combined cardiovascular events in their study after adjusting for age and sex, and LV dyssynchrony was not analyzed in their study. Similarly, we found that LV GLS ≥ −12.94% was associated with cardiovascular events adjusted for age and sex. However, it did not reach statistical significances after adjusting more confounders and in analysis of arrhythmic events in our study. Our study demonstrated that LVLD was an independent risk factor for primary and secondary outcomes, which was also reported in a study evaluated by echocardiography (27). However, the threshold was 45 ms in their study and 89.15 ms in our study. The difference might be ascribed to the fact that we recruited ACM patients with more advanced stage and different imaging technique.

Parameters reflecting RV structural and functional alterations have been revealed to be powerful prognostic risk factors of ACM patients such as RVEF, right ventricular fractional area change (RVFAC), and RV GLS (8, 30, 33). In contrast with previous studies, the present study did not show significant differences of RVEF between ACM patients with and without events. Furthermore, a few studies demonstrated that RV dyssynchrony and GLS were predictors of ventricular arrhythmias in ACM patients (27, 34). The RV GLS, however, was not associated with cardiovascular events in this study. These results could be explained that all ACM patients in our study had advanced stage RV dysfunction, while the previously reported studies enrolled patients at an early stage or preclinical mutation carriers and studied their first adverse events (8, 35). We speculated that the clinical progressions and the risk of events for advanced ACM patients might be more dependent on LV instead of RV performance. Besides, the RV GLS derived from CMR-FT has not been validated in clinical practice as compared with speckle tracking echocardiography (36).

Several limitations should be stressed in this study. First, our study was evaluated in a single tertiary referral center, and was therefore subject to selection bias by including a highly selected population of ACM patients with advanced RV dysfunction or even biventricular dysfunction. Second, the follow-up period was relatively short to enable observation of robust outcome results. Thus, further validation of our results in studies containing larger sample size and longer follow-up duration might be warranted. Third, molecular genetic analysis was not included in this study.

## Conclusions

In this study, we evaluated LV mechanics by CMR-FT technique and highlighted its potential prognostic value in ACM patients in advanced stage. It was demonstrated that LVLD ≥ 89.15 ms assessed by CMR-FT was an independent risk factor for cardiovascular and arrhythmic events, however, the prognostic value of LV GLS and LV GCS was not fully confirmed in this study. CMR-FT derived LV longitudinal dyssynchrony could provide prognostic value for advanced ACM. However, the results of this exploratory analysis should be confirmed by future studies.

## Data Availability Statement

The raw data supporting the conclusions of this article will be made available by the authors, without undue reservation.

## Ethics Statement

The studies involving human participants were reviewed and approved by the Fuwai Hospital Institutional Review Board. The patients/participants provided their written informed consent to participate in this study.

## Author Contributions

YS, LL, LC, and SZ made contributions to conception and design of study. YS drafted the manuscript and collected conventional CMR data. LL and LC were responsible for statistical analysis of the data. LC and RH made critical revisions of the manuscript. XC and KJ were in charge of post processing of CMR-FT analysis. ML and SZ made contribution to study conduction. All authors have read and approved the final manuscript.

## Funding

This study was supported by Grant Nos. 81930044, 81620108015, and 82100377 from the key projects of National Natural Science Foundation of China.

## Conflict of Interest

The authors declare that the research was conducted in the absence of any commercial or financial relationships that could be construed as a potential conflict of interest.

## Publisher's Note

All claims expressed in this article are solely those of the authors and do not necessarily represent those of their affiliated organizations, or those of the publisher, the editors and the reviewers. Any product that may be evaluated in this article, or claim that may be made by its manufacturer, is not guaranteed or endorsed by the publisher.

## Abbreviations

AHA: American Heart Association
ACM: Arrhythmogenic cardiomyopathy
AV: Atrio-ventricular
CI: Confidence interval
CMR: Cardiac magnetic resonance
EF: Ejection fraction
EDVi: End-diastolic volume index
ESVi: End-systolic volume index
FT: Feature tracking
GLS: Global longitudinal strain
ICC: Intraclass correlation coefficient
ICD: Implantable cardioverter defibrillator
LBBB: Left bundle branch block
LGE: Late gadolinium enhancement
LV: Left ventricular
LVLD: Left ventricular longitudinal dyssynchrony
NSVT: Non-sustained ventricular tachycardia
NYHA: New York Heart Association
RBBB: Right bundle branch block
PVC: Premature ventricular complexes
ROC: Receiver operating characteristic
rTFC: Revised task force criteria
RV: Right ventricular
SCD: Sudden cardiac death
SVT: Sustained ventricular tachycardia.
