# Corrigendum: Left Ventricular Longitudinal Dyssynchrony by CMR Feature Tracking Is Related to Adverse Prognosis in Advanced Arrhythmogenic Cardiomyopathy

**DOI:** 10.3389/fcvm.2022.923294

**Published:** 2022-05-04

**Authors:** Yanyan Song, Lu Li, Xiuyu Chen, Keshan Ji, Minjie Lu, Richard Hauer, Liang Chen, Shihua Zhao

**Affiliations:** ^1^Department of Magnetic Resonance Imaging, Fuwai Hospital, National Center for Cardiovascular Diseases, Chinese Academy of Medical Sciences and Peking Union Medical College, Beijing, China; ^2^Department of Diagnostic Radiology, National Cancer Center/National Clinical Research Center for Cancer/Cancer Hospital, Chinese Academy of Medical Sciences and Peking Union Medical College, Beijing, China; ^3^Netherlands Heart Institute and Department of Cardiology, University Medical Center, Utrecht, Netherlands; ^4^Department of Cardiac Surgery, Fuwai Hospital, National Center for Cardiovascular Diseases, Chinese Academy of Medical Sciences and Peking Union Medical College, Beijing, China

**Keywords:** arrhythmogenic cardiomyopathy, magnetic resonance imaging, feature tracking, dyssynchrony, prognosis

**Author Lu Li** contributed equally to this work and act as a co-first author in the published article. The corrected Author Contributions Statement appears below.

**Updated Author Contributions Statement**

YS, LL, LC, and SZ made contributions to conception and design of study. YS drafted the manuscript and collected conventional CMR data. LL and LC were responsible for statistical analysis of the data. LC and RH made critical revisions of the manuscript. XC and KJ were in charge of post processing of CMR-FT analysis. ML and SZ made contribution to study conduction. All authors have read and approved the final manuscript.

The authors apologize for this error and state that this does not change the scientific conclusions of the article in any way. The original article has been updated.

## Publisher's Note

All claims expressed in this article are solely those of the authors and do not necessarily represent those of their affiliated organizations, or those of the publisher, the editors and the reviewers. Any product that may be evaluated in this article, or claim that may be made by its manufacturer, is not guaranteed or endorsed by the publisher.

